# (*E*)-Ethyl *N*′-(3,4,5-trimethoxy­benzyl­idene)hydrazinecarboxyl­ate

**DOI:** 10.1107/S1600536808033461

**Published:** 2008-10-18

**Authors:** Lu-Ping Lv, Xiao-Ming Ding, Wen-Bo Yu, Wei-Wei Li, Xian-Chao Hu

**Affiliations:** a Department of Chemical Engineering, Hangzhou Vocational and Technical College, Hangzhou 310018, People’s Republic of China; b Research Center of Analysis and Measurement, Zhejiang University of Technology, Hangzhou 310014, People’s Republic of China

## Abstract

The mol­ecule of the title compound, C_13_H_18_N_2_O_5_, adopts a *trans* configuration with respect to the C=N bond. The dihedral angle between the benzene ring and the hydrazinecarboxylic acid plane is 49.75 (5)° and an intra­molecular C—H⋯O inter­action occurs. In the crystal structure, the mol­ecules are linked into a chain along [010] by N—H⋯O hydrogen bonds, and a C—H⋯π contact further stabilizes the structure.

## Related literature

For general background, see: Parashar *et al.* (1988 ▶); Hadjoudis *et al.* (1987 ▶). For a related structure, see: Shang *et al.* (2007 ▶).
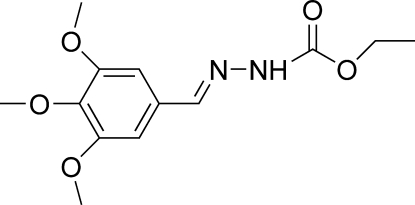


## Experimental

### 

#### Crystal data

C_13_H_18_N_2_O_5_
*M* *_r_* = 282.29
Monoclinic, 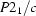

*a* = 22.037 (2) Å
*b* = 4.8782 (5) Å
*c* = 14.0212 (15) Å
β = 108.239 (4)°
*V* = 1431.5 (3) Å^3^
*Z* = 4
Mo *K*α radiation
μ = 0.10 mm^−1^
*T* = 273 (2) K
0.26 × 0.24 × 0.22 mm

#### Data collection

Bruker SMART CCD diffractometer
Absorption correction: multi-scan (*SADABS*; Bruker, 2002 ▶) *T*_min_ = 0.965, *T*_max_ = 0.968
14449 measured reflections
2520 independent reflections
2108 reflections with *I* > 2σ(*I*)
*R*_int_ = 0.029

#### Refinement

*R*[*F* ^2^ > 2σ(*F* ^2^)] = 0.035
*wR*(*F* ^2^) = 0.094
*S* = 1.03
2520 reflections
186 parameters
H-atom parameters constrained
Δρ_max_ = 0.17 e Å^−3^
Δρ_min_ = −0.13 e Å^−3^

### 

Data collection: *SMART* (Bruker, 2002 ▶); cell refinement: *SAINT* (Bruker, 2002 ▶); data reduction: *SAINT*; program(s) used to solve structure: *SHELXS97* (Sheldrick, 2008 ▶); program(s) used to refine structure: *SHELXL97* (Sheldrick, 2008 ▶); molecular graphics: *SHELXTL* (Sheldrick, 2008 ▶); software used to prepare material for publication: *SHELXTL*.

## Supplementary Material

Crystal structure: contains datablocks I, global. DOI: 10.1107/S1600536808033461/hb2820sup1.cif

Structure factors: contains datablocks I. DOI: 10.1107/S1600536808033461/hb2820Isup2.hkl

Additional supplementary materials:  crystallographic information; 3D view; checkCIF report

## Figures and Tables

**Table 1:** Hydrogen-bond geometry (Å, °) *Cg*1 is the centroid of the C4–C9 ring.

*D*—H⋯*A*	*D*—H	H⋯*A*	*D*⋯*A*	*D*—H⋯*A*
N2—H2⋯O4^i^	0.86	1.98	2.8167 (17)	165
C2—H2*A*⋯O1	0.96	2.53	3.062 (2)	115
C1—H1*C*⋯*Cg*1^i^	0.96	2.92	3.755 (2)	146

Symmetry code: (i) 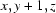.

**Fig. 1.**
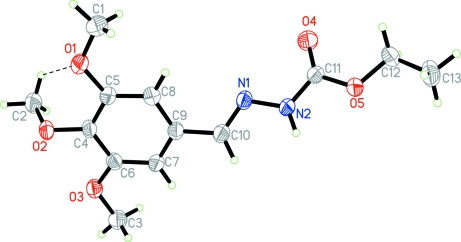
The molecular structure of (I), showing 40% probability displacement ellipsoids for the non-hydrogen atoms. The C—H···O interaction is shown as a dashed line.

**Fig. 2.**
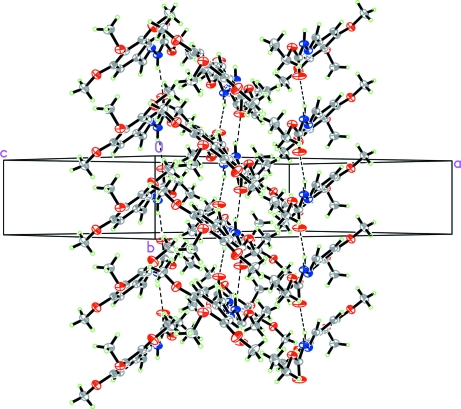
Crystal packing of the title compound, viewed approximately down the a axis. Dashed lines indicate intermolecular hydrogen bonds.

## supplementary crystallographic information

### Comment

Benzaldehydehydrazone derivatives are of interest due to their pharmacological activity (Parashar *et al.*, 1988) and photochromic properties (Hadjoudis *et al.*, 1987). As part of our studies of this type of derivatives, we report herein the crystal structure of the title compound (I).

The title molecule (Fig. 1) adopts a trans configuration with respect to the C═N bond. The hydrazine carboxylic acid methyl ester group is slightly twisted away from the attached ring. The dihedral angle between the benzene ring and the C11/C12//N1/N2/O4/O5 plane is 49.75 (5)°. The bond lengths and angles agree with those observed for (E)-Methyl N'-(4-hydroxybenzylidene)hydrazinecarboxylate (Shang *et al.*, 2007).

The molecules are linked into a chain along [010] by intermolecular and intramolecular N—H···O, C—H···O hydrogen bonds (Fig. 2, Table 1) and a C—H···π contact further stabilizes the structure.

### Experimental

3,4,5-trimethoxybenzaldehyde (1.96 g, 0.01 mol) and methyl hydrazinecarboxylate (1.04 g, 0.01 mol) were dissolved in stirred methanol (25 ml) and left for 3 h at room temperature. The resulting solid was filtered off and recrystallized from ethanol to give the title compound in 90% yield.  Colourless blocks of (I) were obtained by slow evaporation of a ethanol solution at room temperature (m.p. 452–454 K).

### Refinement

The H atoms were geometrically placed (N—H = 0.86 Å, C—H = 0.93–0.96 Å) and refined as riding with U_iso_(H) = 1.2U_eq_(C,N) or 1.5U_eq_(methyl C).

### Crystal data

**Table:**

C_13_H_18_N_2_O_5_	*F*(000) = 600
*M**_r_* = 282.29	*D*_x_ = 1.310 Mg m^−^^3^
Monoclinic, *P*2_1_/*c*	Mo *K*α radiation, λ = 0.71073 Å
Hall symbol: -P 2ybc	Cell parameters from 2520 reflections
*a* = 22.037 (2) Å	θ = 1.0–25.0°
*b* = 4.8782 (5) Å	µ = 0.10 mm^−^^1^
*c* = 14.0212 (15) Å	*T* = 273 K
β = 108.239 (4)°	Block, colourless
*V* = 1431.5 (3) Å^3^	0.26 × 0.24 × 0.22 mm
*Z* = 4	

### Data collection

**Table:**

Bruker SMART CCD diffractometer	2520 independent reflections
Radiation source: fine-focus sealed tube	2108 reflections with *I* > 2σ(*I*)
graphite	*R*_int_ = 0.029
ω scans	θ_max_ = 25.1°, θ_min_ = 1.0°
Absorption correction: multi-scan (SADABS; Bruker, 2002)	*h* = −24→25
*T*_min_ = 0.965, *T*_max_ = 0.968	*k* = −5→5
14449 measured reflections	*l* = −16→16

### Refinement

**Table:**

Refinement on *F*^2^	Secondary atom site location: difference Fourier map
Least-squares matrix: full	Hydrogen site location: inferred from neighbouring sites
*R*[*F*^2^ > 2σ(*F*^2^)] = 0.035	H-atom parameters constrained
*wR*(*F*^2^) = 0.094	*w* = 1/[σ^2^(*F*_o_^2^) + (0.0431*P*)^2^ + 0.3563*P*] where *P* = (*F*_o_^2^ + 2*F*_c_^2^)/3
*S* = 1.03	(Δ/σ)_max_ < 0.001
2520 reflections	Δρ_max_ = 0.17 e Å^−^^3^
186 parameters	Δρ_min_ = −0.13 e Å^−^^3^
0 restraints	Extinction correction: SHELXL97 (Sheldrick, 2008), Fc^*^=kFc[1+0.001xFc^2^λ^3^/sin(2θ)]^-1/4^
Primary atom site location: structure-invariant direct methods	Extinction coefficient: 0.0159 (15)

### Special details

**Table:**

Geometry. All esds (except the esd in the dihedral angle between two l.s. planes) are estimated using the full covariance matrix. The cell esds are taken into account individually in the estimation of esds in distances, angles and torsion angles; correlations between esds in cell parameters are only used when they are defined by crystal symmetry. An approximate (isotropic) treatment of cell esds is used for estimating esds involving l.s. planes.
Refinement. Refinement of F^2^ against ALL reflections. The weighted R-factor wR and goodness of fit S are based on F^2^, conventional R-factors R are based on F, with F set to zero for negative F^2^. The threshold expression of F^2^ > 2sigma(F^2^) is used only for calculating R-factors(gt) etc. and is not relevant to the choice of reflections for refinement. R-factors based on F^2^ are statistically about twice as large as those based on F, and R- factors based on ALL data will be even larger.

### Fractional atomic coordinates and isotropic or equivalent isotropic displacement parameters (Å^2^)

**Table:**

	*x*	*y*	*z*	*U*_iso_*/*U*_eq_	
C7	0.19238 (7)	0.8025 (3)	0.61046 (10)	0.0417 (3)	
H7	0.1961	0.9206	0.6641	0.050*	
C9	0.24447 (7)	0.6457 (3)	0.60759 (10)	0.0399 (3)	
C4	0.13069 (7)	0.6200 (3)	0.44959 (10)	0.0407 (3)	
C8	0.23981 (7)	0.4730 (3)	0.52645 (10)	0.0446 (4)	
H8	0.2743	0.3643	0.5255	0.054*	
C6	0.13492 (7)	0.7831 (3)	0.53350 (10)	0.0400 (3)	
C11	0.44480 (7)	0.3407 (3)	0.82320 (10)	0.0423 (3)	
C5	0.18340 (7)	0.4648 (3)	0.44715 (10)	0.0439 (4)	
C10	0.30489 (7)	0.6728 (3)	0.68892 (10)	0.0424 (3)	
H10	0.3141	0.8365	0.7245	0.051*	
C3	0.08165 (8)	1.0795 (4)	0.61651 (12)	0.0587 (4)	
H3A	0.0958	0.9706	0.6764	0.088*	
H3B	0.0396	1.1488	0.6086	0.088*	
H3C	0.1105	1.2300	0.6215	0.088*	
C12	0.55019 (7)	0.2692 (4)	0.93180 (12)	0.0548 (4)	
H12A	0.5554	0.1264	0.8869	0.066*	
H12B	0.5409	0.1838	0.9881	0.066*	
C2	0.03622 (8)	0.3905 (3)	0.35788 (13)	0.0584 (4)	
H2A	0.0603	0.2298	0.3535	0.088*	
H2B	0.0004	0.4072	0.2979	0.088*	
H2C	0.0212	0.3755	0.4150	0.088*	
C1	0.22489 (9)	0.1433 (4)	0.35569 (13)	0.0716 (6)	
H1A	0.2618	0.2520	0.3588	0.107*	
H1B	0.2117	0.0432	0.2936	0.107*	
H1C	0.2353	0.0171	0.4110	0.107*	
C13	0.60936 (8)	0.4386 (4)	0.96774 (14)	0.0681 (5)	
H13A	0.6185	0.5183	0.9112	0.102*	
H13B	0.6445	0.3248	1.0044	0.102*	
H13C	0.6031	0.5816	1.0108	0.102*	
O5	0.49926 (5)	0.4522 (2)	0.87979 (8)	0.0497 (3)	
O2	0.07592 (5)	0.6268 (2)	0.36863 (7)	0.0485 (3)	
O3	0.08020 (5)	0.9148 (2)	0.53188 (7)	0.0511 (3)	
O1	0.17482 (5)	0.3161 (3)	0.36105 (8)	0.0617 (3)	
O4	0.43507 (6)	0.0998 (2)	0.81180 (10)	0.0752 (4)	
N2	0.40242 (6)	0.5408 (2)	0.78322 (9)	0.0447 (3)	
H2	0.4111	0.7079	0.8021	0.054*	
N1	0.34512 (6)	0.4774 (2)	0.71207 (8)	0.0425 (3)	

### Atomic displacement parameters (Å^2^)

**Table:**

	*U*^11^	*U*^22^	*U*^33^	*U*^12^	*U*^13^	*U*^23^
C7	0.0445 (8)	0.0412 (8)	0.0355 (7)	−0.0013 (6)	0.0070 (6)	−0.0019 (6)
C9	0.0396 (8)	0.0395 (8)	0.0365 (7)	−0.0036 (6)	0.0062 (6)	0.0028 (6)
C4	0.0411 (8)	0.0409 (8)	0.0333 (7)	0.0000 (6)	0.0020 (6)	0.0044 (6)
C8	0.0439 (8)	0.0452 (8)	0.0416 (8)	0.0037 (7)	0.0087 (7)	0.0005 (6)
C6	0.0402 (8)	0.0389 (8)	0.0390 (7)	0.0016 (6)	0.0093 (6)	0.0043 (6)
C11	0.0454 (9)	0.0363 (8)	0.0398 (7)	−0.0023 (6)	0.0055 (6)	−0.0018 (6)
C5	0.0495 (9)	0.0442 (8)	0.0338 (7)	0.0021 (7)	0.0071 (6)	−0.0012 (6)
C10	0.0418 (8)	0.0418 (8)	0.0402 (7)	−0.0041 (7)	0.0077 (6)	−0.0022 (6)
C3	0.0558 (10)	0.0653 (11)	0.0527 (9)	0.0110 (8)	0.0137 (8)	−0.0094 (8)
C12	0.0469 (9)	0.0577 (10)	0.0524 (9)	0.0147 (8)	0.0048 (7)	0.0007 (8)
C2	0.0537 (10)	0.0530 (10)	0.0546 (9)	−0.0050 (8)	−0.0031 (8)	0.0000 (8)
C1	0.0766 (13)	0.0769 (13)	0.0560 (10)	0.0208 (10)	0.0130 (9)	−0.0177 (9)
C13	0.0409 (9)	0.0941 (14)	0.0626 (10)	0.0069 (9)	0.0064 (8)	−0.0054 (10)
O5	0.0398 (6)	0.0418 (6)	0.0562 (6)	0.0018 (4)	−0.0014 (5)	−0.0038 (5)
O2	0.0477 (6)	0.0469 (6)	0.0386 (5)	0.0012 (5)	−0.0043 (4)	0.0037 (4)
O3	0.0443 (6)	0.0595 (7)	0.0434 (5)	0.0111 (5)	0.0048 (5)	−0.0053 (5)
O1	0.0615 (7)	0.0723 (8)	0.0408 (6)	0.0176 (6)	0.0010 (5)	−0.0162 (5)
O4	0.0734 (8)	0.0320 (6)	0.0930 (9)	−0.0032 (6)	−0.0131 (7)	−0.0022 (6)
N2	0.0404 (7)	0.0334 (6)	0.0491 (7)	−0.0041 (5)	−0.0020 (6)	−0.0040 (5)
N1	0.0397 (7)	0.0410 (7)	0.0396 (6)	−0.0060 (5)	0.0019 (5)	−0.0008 (5)

### Geometric parameters (Å, °)

**Table:**

C7—C6	1.3862 (19)	C3—H3B	0.9600
C7—C9	1.391 (2)	C3—H3C	0.9600
C7—H7	0.9300	C12—O5	1.4409 (17)
C9—C8	1.393 (2)	C12—C13	1.492 (2)
C9—C10	1.4630 (19)	C12—H12A	0.9700
C4—O2	1.3744 (16)	C12—H12B	0.9700
C4—C5	1.396 (2)	C2—O2	1.4264 (19)
C4—C6	1.399 (2)	C2—H2A	0.9600
C8—C5	1.385 (2)	C2—H2B	0.9600
C8—H8	0.9300	C2—H2C	0.9600
C6—O3	1.3602 (17)	C1—O1	1.409 (2)
C11—O4	1.1965 (18)	C1—H1A	0.9600
C11—O5	1.3303 (17)	C1—H1B	0.9600
C11—N2	1.3457 (18)	C1—H1C	0.9600
C5—O1	1.3697 (17)	C13—H13A	0.9600
C10—N1	1.2726 (18)	C13—H13B	0.9600
C10—H10	0.9300	C13—H13C	0.9600
C3—O3	1.4250 (18)	N2—N1	1.3772 (16)
C3—H3A	0.9600	N2—H2	0.8600
			
C6—C7—C9	120.07 (13)	O5—C12—H12A	110.4
C6—C7—H7	120.0	C13—C12—H12A	110.4
C9—C7—H7	120.0	O5—C12—H12B	110.4
C7—C9—C8	120.37 (13)	C13—C12—H12B	110.4
C7—C9—C10	119.07 (13)	H12A—C12—H12B	108.6
C8—C9—C10	120.51 (13)	O2—C2—H2A	109.5
O2—C4—C5	121.02 (12)	O2—C2—H2B	109.5
O2—C4—C6	119.39 (13)	H2A—C2—H2B	109.5
C5—C4—C6	119.49 (12)	O2—C2—H2C	109.5
C5—C8—C9	119.44 (14)	H2A—C2—H2C	109.5
C5—C8—H8	120.3	H2B—C2—H2C	109.5
C9—C8—H8	120.3	O1—C1—H1A	109.5
O3—C6—C7	124.73 (13)	O1—C1—H1B	109.5
O3—C6—C4	115.43 (12)	H1A—C1—H1B	109.5
C7—C6—C4	119.84 (13)	O1—C1—H1C	109.5
O4—C11—O5	124.87 (14)	H1A—C1—H1C	109.5
O4—C11—N2	125.79 (14)	H1B—C1—H1C	109.5
O5—C11—N2	109.33 (12)	C12—C13—H13A	109.5
O1—C5—C8	124.38 (14)	C12—C13—H13B	109.5
O1—C5—C4	114.98 (12)	H13A—C13—H13B	109.5
C8—C5—C4	120.61 (13)	C12—C13—H13C	109.5
N1—C10—C9	121.50 (13)	H13A—C13—H13C	109.5
N1—C10—H10	119.2	H13B—C13—H13C	109.5
C9—C10—H10	119.2	C11—O5—C12	117.58 (12)
O3—C3—H3A	109.5	C4—O2—C2	114.74 (11)
O3—C3—H3B	109.5	C6—O3—C3	117.63 (11)
H3A—C3—H3B	109.5	C5—O1—C1	117.99 (12)
O3—C3—H3C	109.5	C11—N2—N1	119.88 (12)
H3A—C3—H3C	109.5	C11—N2—H2	120.1
H3B—C3—H3C	109.5	N1—N2—H2	120.1
O5—C12—C13	106.72 (14)	C10—N1—N2	115.09 (12)
			
C6—C7—C9—C8	1.5 (2)	C7—C9—C10—N1	153.39 (14)
C6—C7—C9—C10	179.09 (13)	C8—C9—C10—N1	−29.0 (2)
C7—C9—C8—C5	1.9 (2)	O4—C11—O5—C12	−0.4 (2)
C10—C9—C8—C5	−175.66 (13)	N2—C11—O5—C12	178.13 (13)
C9—C7—C6—O3	175.98 (13)	C13—C12—O5—C11	166.29 (13)
C9—C7—C6—C4	−4.5 (2)	C5—C4—O2—C2	77.45 (18)
O2—C4—C6—O3	7.15 (19)	C6—C4—O2—C2	−106.16 (16)
C5—C4—C6—O3	−176.39 (13)	C7—C6—O3—C3	−1.6 (2)
O2—C4—C6—C7	−172.45 (13)	C4—C6—O3—C3	178.83 (14)
C5—C4—C6—C7	4.0 (2)	C8—C5—O1—C1	5.2 (2)
C9—C8—C5—O1	175.76 (14)	C4—C5—O1—C1	−176.66 (15)
C9—C8—C5—C4	−2.3 (2)	O4—C11—N2—N1	−9.3 (2)
O2—C4—C5—O1	−2.5 (2)	O5—C11—N2—N1	172.11 (11)
C6—C4—C5—O1	−178.86 (13)	C9—C10—N1—N2	173.88 (12)
O2—C4—C5—C8	175.78 (13)	C11—N2—N1—C10	170.86 (13)
C6—C4—C5—C8	−0.6 (2)		

### Hydrogen-bond geometry (Å, °)

**Table:**

*D*—H···*A*	*D*—H	H···*A*	*D*···*A*	*D*—H···*A*
N2—H2···O4^i^	0.86	1.98	2.8167 (17)	165
C2—H2A···O1	0.96	2.53	3.062 (2)	115
C1—H1C···Cg1^i^	0.96	2.92	3.755 (2)	146

Symmetry codes: (i) *x*, *y*+1, *z*.
